# Effects of Marine Protein Hydrolysates as Squid-Liver Powder Replacements on Growth Performance, Digestive Capacity and Health Status of Pacific White Shrimp (*Litopenaeus vannamei*)

**DOI:** 10.3390/ani16091415

**Published:** 2026-05-06

**Authors:** Dachawat Poonnual, Siriporn Tola, Bundit Yuangsoi

**Affiliations:** Department of Fisheries, Faculty of Agriculture, Khon Kaen University, Khon Kaen 40002, Thailand; dachawat.poonnual@symrise.com (D.P.);

**Keywords:** Pacific white shrimp (*Litopenaeus vannamei*), marine protein hydrolysates, growth performance, feed utilization, digestive enzyme activity, health status

## Abstract

This study investigated the replacement of squid-liver powder (SLP) with different marine protein hydrolysates in diets for Pacific white shrimp (*Litopenaeus vannamei*) over an eight-week feeding period. A control diet contained 5% SLP, whereas four test diets replaced 1% of the diet (dry-matter basis) with tuna hydrolysate liquid (TH), shrimp hydrolysate powder (SH), fish hydrolysate powder (FH), or salmon silage liquid (SS), with soybean meal adjusted to maintain dietary protein balance. Shrimp fed the TH diet showed higher final body weight (15.76 ± 0.24 g shrimp^−1^) and specific growth rate (3.18 ± 0.02% day^−1^) than those fed the other experimental diets (*p* < 0.05). TH also increased feed intake (20.96 ± 0.49 g shrimp^−1^) and was associated with higher trypsin activity (3236 ± 226 unit h^−1^ mg protein^−1^) and improved protein digestibility. Histological observations indicated greater hepatopancreatic cell density and increased intestinal villus height in the TH group. In addition, survival of shrimp fed TH (90.0 ± 2.8%) was higher than that observed in the FH and SS groups (*p* < 0.05), and hemolymph from TH-fed shrimp showed stronger inhibitory activity against *Vibrio parahaemolyticus*. Overall, among the evaluated hydrolysates, 1% tuna hydrolysate (dry-matter basis) produced the most consistent improvements across growth performance, digestive capacity, and selected health-related indicators, supporting its feasibility as a partial substitute for squid liver powder in shrimp diets.

## Simple Summary

Ingredient safety and feed efficiency are key challenges in shrimp farming, particularly with the long-term use of squid-based products, which may raise concerns about cadmium accumulation. This study evaluated marine protein hydrolysates as functional alternatives in Pacific white shrimp diet. Tuna hydrolysate liquid at 1% (dry matter) inclusion significantly improved growth, feed utilization, digestive capacity, intestinal structure, and resistance to bacterial challenge. These results indicate that tuna hydrolysate liquid can effectively replace squid-liver powder while supporting sustainable and efficient shrimp production.

## 1. Introduction

Shrimp aquaculture has expanded rapidly over the past decade, with global production of Pacific white shrimp (*Litopenaeus vannamei*) reaching 6.8 million tons in 2022, accounting for approximately 62.2% of total shrimp production worldwide [1]. However, market oversupply has depressed shrimp prices, compelling farmers to sustain production while improving economic efficiency. As feed costs represent more than 50% of total production expenses in intensive systems, optimizing feed efficiency remains a key approach for both cost control and environmental sustainability [2,3,4].

Marine-derived protein ingredients, particularly fish meal, shrimp meal, and squid-based products, remain essential components of aquafeeds due to their high palatability and nutritional quality. Among these, squid-derived ingredients such as squid meal, squid-liver powder (SLP), and squid viscera meal are widely incorporated as feeding stimulants to enhance feed intake and growth performance in a variety of aquatic species [2,5,6,7,8]. Squid-liver powder, produced from squid processing by-products (e.g., heads and viscera), has long been utilized in shrimp feeds to improve feeding response and nutrient utilization and remains commercially available and commonly applied in the aquafeed industry [4,9,10].

Despite its functional benefits, the use of squid-based ingredients involves several practical considerations that warrant continued evaluation. In Pacific white shrimp (*L. vannamei*), moderate inclusion levels of squid meal have been reported to enhance growth and digestive enzyme activity, whereas excessive inclusion may adversely affect performance [7,11]. Moreover, previous studies have indicated that squid processing by-products, particularly viscera meals, can contain elevated levels of certain heavy metals, especially cadmium, which is known to accumulate in marine organisms and seafood by-products [8,10,12]. Cadmium is a toxic element with a long biological half-life and has been associated with adverse physiological effects in aquatic organisms, including oxidative stress and tissue damage [13,14,15]. Importantly, these concerns are derived from findings reported in earlier studies and are presented here as contextual background reflecting broader considerations related to ingredient selection, rather than as outcomes directly evaluated in the present investigation.

Accordingly, there is increasing interest in identifying alternative marine-derived protein ingredients that can provide comparable functional performance while supporting more consistent quality, improved safety perception, and better utilization of seafood processing by-products. Marine protein hydrolysates, produced via enzymatic hydrolysis of proteins into low-molecular-weight peptides and free amino acids, often from seafood processing residues representing more than 60% of raw material streams, have emerged as promising functional ingredients in aquafeeds [16,17,18]. These hydrolysates are characterized by high digestibility, balanced amino acid profiles, and readily bioavailable nutrients. Across a wide range of aquatic species, dietary inclusion of marine protein hydrolysates (typically 1–10%) has been shown to enhance feed utilization, growth performance, nutrient digestibility, and non-specific immune responses, including in Asian seabass (*Lates calcarifer*) [19,20,21,22,23,24], European seabass (*Dicentrarchus labrax*) [25], red seabream (*Pagrus major*) [26], olive flounder (*Paralichthys olivaceus*) [26,27,28,29], African catfish (*Clarias gariepinus*) [30], Nile tilapia (*Oreochromis niloticus*) [31], snakehead fish (*Channa striata*) [32], and Pacific white shrimp [33,34]. Notably, Gunathilaka et al. [33] demonstrated that approximately 1% tuna hydrolysate liquid (TH) or shrimp hydrolysate powder (SH) could replace 5% SLP in shrimp diets without compromising growth performance. However, that study did not examine effects on digestive enzyme activity, histological characteristics, or immune-related responses, leaving important gaps in understanding the broader physiological implications of such substitutions.

Therefore, the present study aimed to evaluate the effects of replacing squid-liver powder with alternative marine protein hydrolysates—tuna hydrolysate liquid (TH), shrimp hydrolysate powder (SH), fish hydrolysate powder (FH), and salmon silage liquid (SS)—in diets for Pacific white shrimp. This study provides a comprehensive assessment of growth performance, feed utilization, digestive enzyme activity, nutrient digestibility, immune-related parameters, and histological characteristics of the hepatopancreas and intestine.

## 2. Materials and Methods

### 2.1. Experimental Diets

Five isonitrogenous (44% crude protein), isolipidic (9% crude lipid), and isocaloric (19 MJ kg^−1^) experimental diets were formulated. The marine ingredients—squid liver powder (SLP), tuna hydrolysate liquid (TH), shrimp hydrolysate powder (SH), fish hydrolysate powder (FH), and salmon silage liquid (SS) and their chemical composition, molecular weight distribution, and amino acid profiles are presented in Table 1. Squid liver powder was produced from squid or cuttlefish by-products; TH was derived from tuna viscera via enzymatic hydrolysis; SH was obtained from shrimp processing waste (35–45% inedible fraction); FH was produced from tilapia and marine fish by-products through enzymatic processing; and SS was prepared from category-3 salmon by-products treated with 2% formic acid and endogenous gut enzymes. All marine ingredients were supplied by Marine and Feed, SPF Diana (Thailand) Co., Ltd. (Symrise Group), Samutsakhon, Thailand.

A diet containing 5% SLP and 10% fish meal (crude basis) served as the control, while four experimental diets were formulated by replacing 1% TH, SH, FH, or SS on a dry-matter basis. Soybean meals were included to compensate for dietary protein, and all diets were balanced to meet essential nutrient requirements [35].

Experimental diets (Table 2) were prepared by thoroughly mixing powdered ingredients with fish oil and soybean oil, followed by the addition of 30% boiling water. The resulting dough was pelletized using a single-screw extruder (Model EXT15HP3V03, Siam Farm Services Co., Ltd., Ko Kha, Thailand) equipped with a 2-mm die, dried at 90 °C for 4 h, and stored at −20 °C until use. Representative diet samples were randomly collected for chemical composition, molecular-weight distribution, and amino acid profile analyses (Table 2 and Table 3).

### 2.2. Experimental Animals and Conditions and Feeding Procedure

The feeding trial was conducted indoors using a closed recirculating saltwater system at the AQUALIS APAC testing center, Marine and Feed, SPF Diana (Thailand) Co., Ltd., Samutsakhon, Thailand. Pacific white shrimp with an initial body weight of 1–2 g were used. Prior to the experiment, shrimp were acclimated for two weeks and fed a commercial diet (Inteqc 113s; 42% crude protein and 8% crude lipid) twice daily. The rearing system consisted of polyethylene tanks connected to a drum filter and a moving bed bioreactor, with a water renewal rate of 300 L h^−1^ and continuous aeration to maintain adequate dissolved oxygen levels. Throughout the feeding trial, water quality parameters were monitored and maintained within optimal ranges for Pacific white shrimp: temperature 29.93 ± 0.65 °C, pH 8.22 ± 0.27, dissolved oxygen 5.06 ± 1.00 mg L^−1^, salinity 29.64 ± 2.12 ppt, ammonia and nitrite 0.0–0.5 mg L^−1^, calcium 227 ± 44 mg L^−1^, magnesium 982 ± 145 mg L^−1^, and alkalinity 179 ± 34 mg L^−1^. A photoperiod of 12 h light and 12 h darkness was maintained using fluorescent lighting.

Fifty shrimp (average initial body weight 2.66 ± 0.01 g shrimp^−1^; mean ± SD, n = 5), determined by bulk weighing, were randomly allocated to twenty-five 500 L polyethylene tanks, corresponding to a stocking density of approximately 80 shrimp m^−2^ or and 125 shrimp m^−3^. Each experimental diet was randomly assigned to five replicate tanks and fed at 10% of tank biomass, divided into three daily meals (08:00, 12:00, and 16:00 h) for eight weeks. Uneaten feed was collected one hour after feeding, oven-dried, and used to estimate feed intake. Feeding rations were subsequently adjusted according to the measured feed consumption.

### 2.3. Sample Collection

At the end of the 8-week feeding trial, shrimp were fasted for 24 h and bulk-weighed to determine final body weight. Prior to sampling, shrimp were anesthetized by immersion in chilled water for 5 min. Two shrimp were randomly selected from each tank (n = 10 per dietary treatment) for hemolymph collection. Hemolymph was withdrawn from the ventral sinus using 1-mL syringes fitted with 23-gauge, 1.5-inch needles preloaded with 500 µL of anticoagulant (10% trisodium citrate in RPMI 1640; Gibco™, Waltham, MA, USA) and mixed with hemolymph at a 1:1 (*v*/*v*) ratio for hematological, immunological, and metabolic marker analyses. For serum collection, hemolymph samples were centrifuged at 3500 rpm for 5 min to separate the serum, which was subsequently stored at −80 °C until analysis. The hepatopancreas was excised, immediately weighed to calculate the hepatosomatic index (HSI, %), and rapidly frozen at −80 °C for subsequent digestive enzyme activity analyses. An additional two shrimp were randomly selected from each tank (n = 10 per dietary treatment) for hepatopancreas and intestine collection, and the tissues were preserved in Davidson’s fixative for histological analysis.

### 2.4. Analytical Methods

#### 2.4.1. Growth Performance, Feed Utilization and Survival Rate

Measurements at this stage were for calculating growth performance, feed utilization and survival rate using the following equations:

Specific growth rates (SGR, % day^−1^) = 100 × [(ln final body weight (g shrimp^−1^) − In initial body weight (g shrimp^−1^))/day of feeding (day)]

Feed conversion ratio (FCR) = dry feed intake (g shrimp^−1^)/body weight gain (g shrimp^−1^)

Hepatosomatic index (HSI, %) = 100 × (hepatopancreas weight (g shrimp^−1^)/body weight (g shrimp^−1^))

Survival rate (%) = 100 × [(initial shrimp number (shrimp) − final shrimp number (shrimp)/initial shrimp number (shrimp)]

#### 2.4.2. Hematological and Non-Specific Immunity Assay

Total hemocyte count (THC) was measured after diluting hemolymph (0.02 mL) in Dip-Quick staining solution (0.98 mL), followed by counting with a hemocytometer under phase-contrast microscopy.

Lysozyme activity was assessed using a turbidimetric assay with lyophilized *Micrococcus lysodeikticus* (ATCC 4698; Sigma-Aldrich, St. Louis, MO, USA) as the substrate, based on Parry et al. [37], with minor modifications.

Superoxide dismutase (SOD) and catalase (CAT) activities were determined using commercial assay kits, including the SOD determination kit (Cat. No. 19160; Sigma-Aldrich, Taufkirchen, Germany) and the catalase assay kit (K-CATAL 07/19; Megazyme, Bray, Ireland).

Phenol oxidase (PO) activity was measured spectrophotometrically using L-dihydroxyphenylalanine (Sigma-Aldrich, USA) as the substrate, according to the methods described by Söderhäll and Häll [38] and Hernández-López et al. [39].

Respiratory burst activity was assessed by measuring the reduction of nitroblue tetrazolium (NBT) to blue formazan, following the method adapted from Song and Hsieh [40].

The clearance efficiency of shrimp hemolymph against *Vibrio harveyi* and *Vibrio parahaemolyticus* was evaluated using a modified method described by Kewcharoen and Srisapoome [41], to assess hemolymph clearance efficiency against opportunistic and pathogenic bacteria.

#### 2.4.3. Hemolymph Metabolic Markers Assay

Hemolymph metabolic parameters analyzed in this study included cholesterol (enzymatic, OXI/PER), triglycerides (enzymatic, OXI/PER), albumin (colorimetric, BCG), total protein (colorimetric, biuret), glucose (enzymatic, oxidase), creatinine (enzymatic, Jaffé), aspartate aminotransferase (AST; kinetic, IFCC), and alkaline phosphatase (ALP; kinetic, IFCC). All analyses were performed using an automatic biochemistry analyzer (BA400; BioSystems, Barcelona, Spain).

#### 2.4.4. Digestive Enzyme Assay

Crude enzyme extracts were prepared from the hepatopancreas of shrimp (n = 10) following the method described by Rungruangsak-Torrissen et al. [42]. Hepatopancreas samples were homogenized in 50 mM Tris-HCl buffer (pH 8.0) containing 200 mM NaCl at a ratio of 1:5 (*w*/*v*). The homogenates were centrifuged at 12,000 rpm at 4 °C for 30 min, and the resulting supernatants were collected and stored at −20 °C prior to enzyme activity analyses. Activities of trypsin, chymotrypsin, total protease, amylase, lipase, and total protein were subsequently determined.

Trypsin and chymotrypsin activities were assayed according to Rungruangsak-Torrissen et al. [43]. Briefly, 2 µL of the supernatant was mixed with 200 µL of substrate solution containing either 1.25 mM benzoyl-L-arginine-p-nitroanilide (BAPNA) for trypsin or 0.10 mM N-succinyl-Ala-Ala-Pro-Phe-p-nitroanilide (SAPNA) for chymotrypsin. Enzyme activity was monitored at 405 nm for 1.5 min at 50 °C using a UV/VIS spectrophotometer (Double Beam Spectrophotometer Libra S80, Biochrom Ltd., Cambridge, UK).

Total protease activity was determined following the method of Areekijseree et al. [44], based on azocasein hydrolysis in phosphate buffer (pH 9.0), with absorbance measured at 440 nm after trichloroacetic acid precipitation. Amylase activity was quantified using the 3,5-dinitrosalicylic acid (DNS) method by measuring reducing sugars at 540 nm, with maltose used as the standard.

Lipase activity was determined using p-nitrophenyl palmitate (0.01 M) as the substrate in 0.2 M phosphate buffer (pH 8.0), and absorbance was measured at 410 nm [45].

Total protein content of the enzyme extract was determined using the method of Lowry et al. [46], with bovine serum albumin (BSA) as the standard.

#### 2.4.5. Histological of Hepatopancreas and Intestine Assay

After fixation in Davidson’s fixative for 24 h, histological examinations of the hepatopancreas and intestine were conducted following the method described by Bell and Lightner [47]. Samples were dehydrated through a graded ethanol series of increasing concentrations up to 100% and subsequently embedded in paraffin. Tissue sections (4–5 µm thick) were prepared and stained with hematoxylin and eosin (H&E) for morphological evaluation. Histological images were captured at 10× and 40× magnifications using a digital microscope camera (Olympus, EPview software version V3.7.7_20230531).

### 2.5. Digestibility Analysis

#### 2.5.1. *In Vitro* Protein Digestibility of Ingredients and Diets

*In vitro* protein digestibility of ingredients and diets was assessed based on Rungruangsak-Torrissen et al. [48] and Rungruangsak-Torrissen [43]. A 30 mg protein sample was incubated (200 rpm, 30 °C, 24 h) with phosphate buffer (50 mM, pH 8.2) and chloramphenicol (0.5%), followed by addition of dialyzed crude enzyme extract. Digestion was quantified using a TNBS assay (420 nm) with DL-alanine as standard.

#### 2.5.2. Apparent Digestibility Coefficients (ADCs)

Apparent digestibility coefficients (ADCs), diets were supplemented with 1% Cr_2_O_3_ as an inert marker. Feces were collected by siphoning three times daily, starting after one week of feeding, and processed by filtration, freezing (−20 °C), drying (60 °C overnight), and grinding. Chromium oxide was analyzed following Austreng [49], and ADCs were calculated as described by Cho et al. [50]. Proximate composition of diets and feces was determined using AOAC procedures [51].

### 2.6. Statistical Analysis

Data were analyzed using SPSS (version 23). Normality and homogeneity of variance were checked prior to one-way ANOVA assessing dietary effects on growth, feed utilization, and health-related indices. Tukey’s HSD test was used for post hoc comparisons when significant effects were detected (*p* < 0.05). Results are presented as mean ± SD.

## 3. Results

### 3.1. Growth Performance, Feed Utilization and Survival Rate

Growth outcomes are summarized in Table 4. The TH group achieved greater FBW, and SGR than the other treatments (*p* < 0.05). Feed intake was also higher in the TH group than in SLP and FH groups (*p* < 0.05), whereas FCR was not affected by diet (*p* > 0.05). Survival differed among treatments; the TH group resulted in higher survival than the FH and SS groups (*p* < 0.05), but did not differ from the SLP or SH groups (*p* > 0.05).

### 3.2. Hematological and Non-Specific Immunity

**Figure 1:**
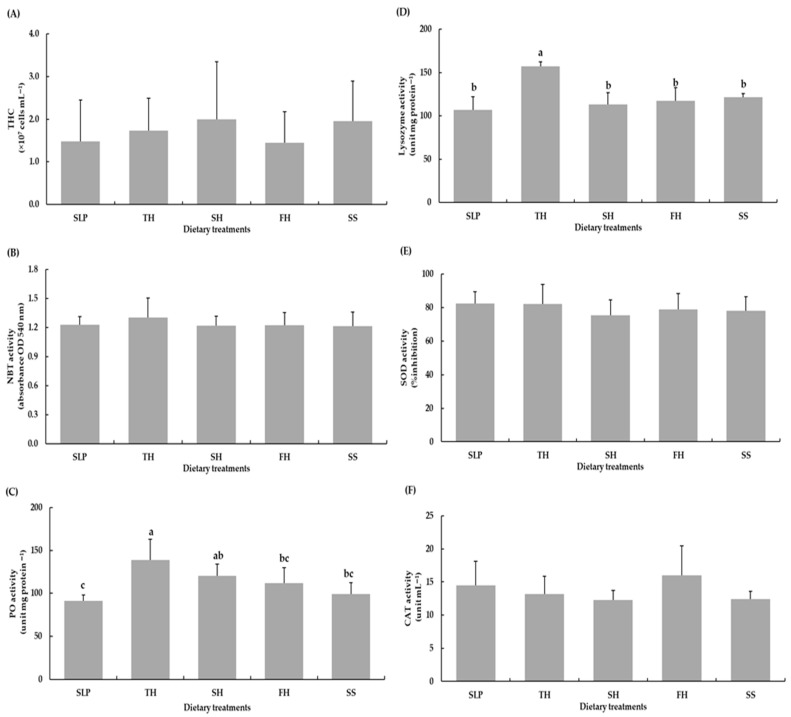
(**A**), Total hemocytes count (THC); (**B**), Nitroblue-tetrazolium activity (NBT); (**C**), Phenol oxidase activity (PO); (**D**), Lysozyme activity; (**E**), Supper oxide dismutase activity (SOD); and (**F**), Catalase activity (CAT) of Pacific white shrimp fed the experimental diets for 8 weeks. Values are shown as means ± SD (*n* = 5). Different letters indicate statistical differences (*p* < 0.05) among the treatments. Abbreviations: SLP, squid-liver powder; TH, tuna hydrolysate liquid; SH, shrimp hydrolysate powder; FH, fish hydrolysate powder; and SS, salmon silage liquid.

Hematological and non-specific immune parameters of shrimp fed the experimental diets, including total hemocyte count (THC), nitroblue tetrazolium (NBT) activity, superoxide dismutase (SOD) activity, and catalase (CAT) activity, did not differ significantly from those of shrimp fed the SLP diet (*p* > 0.05) (Figure 1). In contrast, lysozyme activity in shrimp fed the TH diet was significantly higher than that observed in shrimp fed the SLP or any other experimental diets (*p* < 0.05) (Figure 1D).

**Figure 2:**
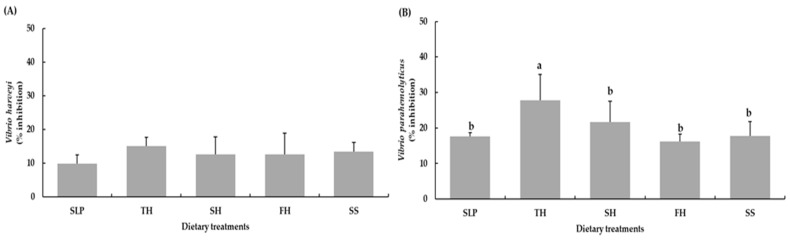
Clearance efficiency of hemolymphs on *Vibrio harveyi* (**A**) and *Vibrio parahaemolyticus* (**B**) against Pacific white shrimp fed the experimental diets for 8 weeks. Values are shown as means ± SD (*n* = 5). Different letters indicate statistical differences (*p* < 0.05) among the treatments. Abbreviations: SLP, squid-liver powder; TH, tuna hydrolysate liquid; SH, shrimp hydrolysate powder; FH, fish hydrolysate powder; and SS, salmon silage liquid.

Furthermore, phenol oxidase (PO) activity was increased in shrimp fed the TH, SH, FH, and SS diets, with significantly higher PO activity compared with shrimp fed the SLP diet (*p* < 0.05) (Figure 1C). Hemolymph clearance efficiency against Vibrio harveyi did not differ significantly among dietary treatments, with inhibition rates ranging from 9.93% to 15.13% (*p* > 0.05). In contrast, hemolymphs from shrimp fed the TH diet exhibited significantly greater inhibitory activity against *Vibrio parahaemolyticus* (27.87%) compared with all other dietary treatments (*p* < 0.05) (Figure 2).

### 3.3. Serum Metabolic Markers

Serum metabolic parameters, including cholesterol, triglycerides, albumin, total protein, glucose, creatinine, aspartate aminotransferase, and alkaline phosphatase, were not significantly affected by the dietary treatments (*p* > 0.05). However, shrimp fed the FH diet exhibited significantly higher hemolymph glucose levels compared with those fed the other experimental diets (*p* < 0.05) (Table 5).

### 3.4. Digestive Enzymes Activity

Digestive enzyme activities in the hepatopancreas of Pacific white shrimp fed the experimental diets for 8 weeks are presented in Table 6. Trypsin activity in the hepatopancreas of shrimp fed the TH diet was significantly higher than that observed in shrimp fed the other experimental diets (*p* < 0.05). In contrast, chymotrypsin, total protease, amylase, and lipase activities did not differ significantly among dietary treatments (*p* > 0.05).

### 3.5. Histological of Hepatopancreas and Intestine

**Figure 3:**
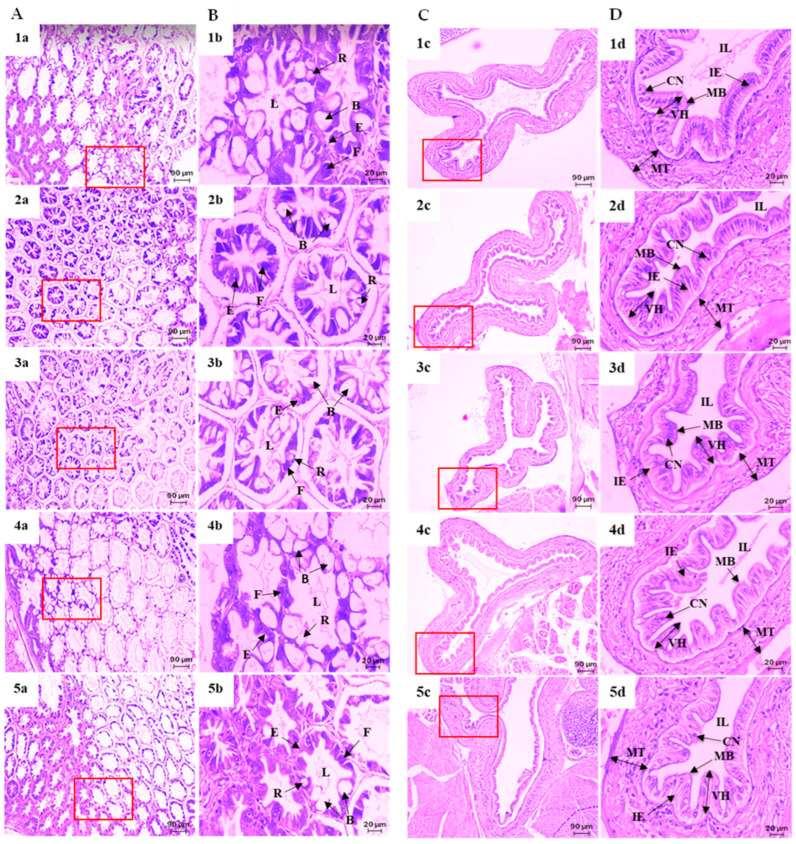
Histological sections of hepatopancreas (**A**,**B**) and intestine (**C**,**D**) of Pacific white shrimp after 8 weeks feeding. Panels: (**1a**–**1d**), SLP diet; (**2a**–**2d**), TH diet; (**3a**–**3d**), SH diet; (**4a**–**4d**), FH diet; and (**5a**–**5d**), SS diet. Images at 10× magnification (**A**,**C**) and 40× magnification (**B**,**D**). Panels (**B**,**D**) were taken from the regions indicated by the red squares in panels (**A**,**C**), respectively. Abbreviations: B, B-cells (blasenzellen); E, E-cells (embryonalzellen or embryonic); F, F-cells (fibrillenzellen or fibrous); L, lumen; R, R-cells (restzellen); CN, cell nuclei; IE, intestinal epithelium; IL, intestinal lumen; VH, villi height; MT, muscle intestinal thickness; MB, mucosa brush border.

Histological examination of the hepatopancreas and intestine of Pacific white shrimp fed the experimental diets for 8 weeks is presented in Figure 3. Shrimp from all dietary treatments exhibited normal hepatopancreatic (HP) architecture with clearly distinguishable cell types, including B-cells (blasenzellen), E-cells (embryonalzellen), F-cells (fibrillenzellen), and R-cells (restzellen) (Figure 3A,B).

**Figure 4:**
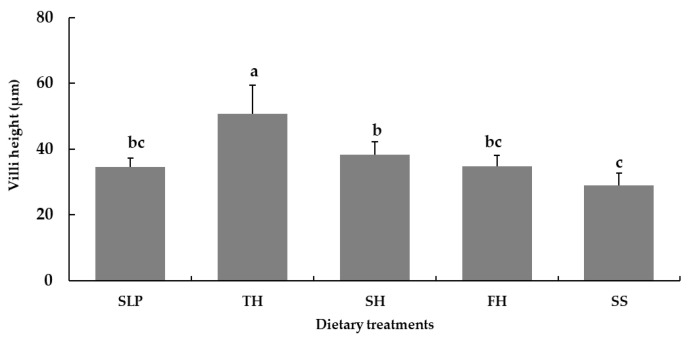
Villi height measurements for Pacific white shrimp that have received experimental diets for 8 weeks. Values are shown as means ± SD (n = 5). Different letters indicate statistical differences (*p* < 0.05) among the treatments. Abbreviations: SLP, squid-liver powder; TH, tuna hydrolysate liquid; SH, shrimp hydrolysate powder; FH, fish hydrolysate powder; and SS, salmon silage liquid.

Intestinal tissues of shrimp fed diets containing marine ingredients exhibited well-organized epithelial structures with tightly connected cells. Intestinal villi height (VH) was significantly greater in shrimp fed the TH diet compared with all other dietary treatments (*p* < 0.05). Moreover, shrimp fed the SH diet exhibited significantly higher VH than those fed the SS diets, although no significant differences were observed when compared with the SLP and FH diets s (*p* > 0.05) (Figure 3 and Figure 4).

### 3.6. Nutrient Digestibility

#### 3.6.1. *In Vitro* Protein Digestibility of Ingredients and Diets

**Figure 5:**
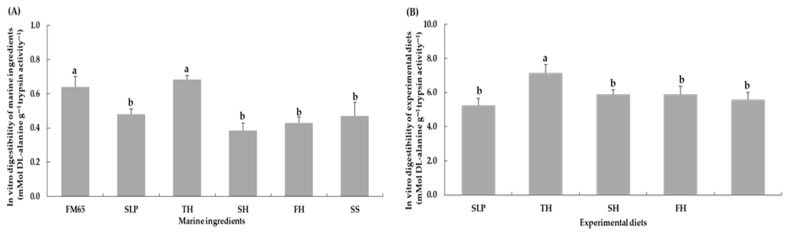
*In vitro* protein digestibility of marine ingredients (**A**) and experimental diets (**B**). Values are presented as means ± SD (*n* = 5). Different letters indicate statistical differences (*p* < 0.05) among the treatments. Abbreviations: FM65, fish meal 65% crude protein; SLP, squid-liver powder; TH, tuna hydrolysate liquid; SH, shrimp hydrolysate powder; FH, fish hydrolysate powder; and SS, salmon silage liquid.

Protein digestibility of marine hydrolysates in the fish meal (65% crude protein) and TH groups (0.64 ± 0.06 and 0.68 ± 0.02 mMol DL-alanine g^−1^ trypsin activity^−1^, respectively) was significantly higher than that observed in the SLP, SH, FH, and SS groups (0.48 ± 0.03, 0.38 ± 0.05, 0.43 ± 0.04, and 0.47 ± 0.08 mMol DL-alanine g^−1^ trypsin activity^−1^, respectively) (*p* < 0.05) (Figure 5A). Moreover, overall protein digestibility of the TH diet (7.16 ± 0.49 mMol DL-alanine g^−1^ trypsin activity^−1^) was the highest among all experimental diets and differed significantly from the other treatments (*p* < 0.05). In contrast, no significant differences were observed among the SLP, SH, FH, and SS diets, with values ranging from 5.26 to 5.91 mMol DL-alanine g^−1^ trypsin activity^−1^ (*p* > 0.05) (Figure 5B).

#### 3.6.2. Apparent Digestibility Coefficients (ADCs)

The apparent digestibility coefficients (ADCs) for dry matter, protein, and lipid in shrimp fed the experimental diets are presented in Table 7. Shrimp fed the TH diet exhibited a significantly higher protein ADC compared with those fed the other diets (*p* < 0.05). Furthermore, shrimp receiving the SH and FH diets showed significantly greater protein ADCs than those fed the SLP and SS diets (*p* < 0.05). In contrast, no significant differences were observed among dietary treatments in the ADCs of dry matter or lipid (*p* > 0.05).

## 4. Discussion

Marine protein hydrolysates are increasingly recognized as functional feed ingredients in aquaculture due to their high proportion of low-molecular-weight peptides, free amino acids, and soluble nitrogen compounds, which contribute to improved palatability, nutrient utilization, and physiological performance in shrimp [33,34]. In the present study, dietary supplementation with tuna hydrolysate liquid (TH) at 1% resulted in consistent improvements in growth performance, feed intake, protein digestibility, and selected immune responses in Pacific white shrimp. These findings align with previous studies reporting beneficial effects of protein hydrolysates on growth and feed utilization across aquatic species [20,21,22,23,24,26,27,28,52,53]. The enhanced growth performance observed in shrimp fed the TH diet appears to be primarily associated with increased feed intake and improved nutrient assimilation. Protein hydrolysates contain high levels of free amino acids and small peptides that function as effective feeding stimulants in crustaceans [54]. Amino acids such as glycine, alanine, arginine, glutamic acid, and taurine are well documented to enhance feed attractability and ingestion in shrimp [54,55]. The amino acid composition of the TH diet in the present study likely contributed to improved palatability, leading to higher feed consumption and consequently greater weight gain.

In addition to feed intake, improved protein digestibility appears to be a key mechanism underlying the enhanced growth performance observed in this study. Shrimp fed the TH diet showed significantly higher protein digestibility, likely due to the high degree of hydrolysis in TH that produces short peptides readily absorbed through intestinal peptide transport systems. Evidence from mammalian studies indicates that PEPT1 efficiently transports a wide range of di- and tripeptides, reflecting its capacity for rapid uptake of amino-nitrogen even under compromised mucosal conditions [56]. Similarly, research in post-larval Senegal sole has demonstrated that free amino acids and small peptides are absorbed faster and with greater assimilation efficiency than intact proteins [57], supporting the concept that peptide-based nitrogen is more efficiently utilized. These mechanisms may help explain the enhanced peptide absorption and improved nitrogen utilization observed in shrimp fed TH, consistent with reports from other aquatic species receiving hydrolyzed protein sources [58,59].

The significant increase in trypsin activity in shrimp fed the TH diet further supports the role of hydrolysates in enhancing digestive capacity [20,22,28,31,50]. In fish, digestive enzyme secretion is influenced by dietary protein composition and amino acid availability through nutrient-sensitive regulatory pathways, including those involving cholecystokinin (CCK) [57,60]. Although crustaceans do not possess an identical regulatory system, they exhibit neuroendocrine responses to dietary nutrients that modulate digestive enzyme activity [61]. The elevated trypsin activity observed here may therefore reflect a physiological response to increased availability of digestible peptides and amino acids in the TH diet. Comparable increases in proteolytic enzyme activity have been reported in both fish and shrimp fed protein hydrolysates [42,60].

Histological observations further corroborated the improvement in digestive capacity, as shrimp fed the TH diet exhibited significantly greater intestinal villus height and enhanced hepatopancreatic cellular density. Increased villus height is commonly associated with a larger absorptive surface area and improved nutrient uptake efficiency [26,62,63,64,65]. Moreover, bioactive peptides derived from protein hydrolysates have been reported to promote intestinal epithelial development and gut health [66]. The improved intestinal morphology observed in the present study may therefore be associated with the presence of low-molecular-weight peptides, which could enhance nutrient absorption and overall growth performance.

From an immunological perspective, dietary TH significantly increased lysozyme activity and enhanced hemolymph clearance efficiency against *Vibrio parahaemolyticus*. These results suggest that protein hydrolysates may exert immunomodulatory effects in shrimp [33,67]. Bioactive peptides derived from enzymatic hydrolysis have been reported to possess antimicrobial and immunostimulatory properties, acting as pathogen-associated molecular pattern (PAMP)-like molecules that activate innate immune responses [68,69]. The observed increase in lysozyme and phenol oxidase activities may therefore reflect activation of non-specific immune pathways, enhancing the shrimp’s capacity to respond to pathogenic challenges [22,25,26,29,34,61,70,71,72]. However, as no in vivo challenge test was conducted, these results should be interpreted as indicators of enhanced immune potential rather than direct evidence of increased disease resistance.

Most hematological and metabolic parameters were not significantly influenced by dietary treatments, suggesting that inclusion of hydrolysates did not induce physiological stress or metabolic imbalance. The higher glucose level observed in shrimp fed the FH diet may reflect alterations in energy metabolism rather than a stress response, as growth and survival were not negatively affected [73,74]. Similar patterns have been reported in shrimp, where fluctuations in hemolymph glucose levels are associated with metabolic adjustments rather than stress conditions [75,76]. Further investigation is required to clarify this response.

Despite the growing interest in plant-based ingredients as sustainable alternatives in aquafeeds, the present results support the continued use of marine-derived functional ingredients in shrimp nutrition. Plant protein sources are often limited by essential amino acid imbalances (e.g., methionine, lysine, and taurine) and anti-nutritional factors, which can impair feed intake, nutrient digestibility, and intestinal health in Pacific white shrimp [5,62,65,71]. In contrast, marine protein hydrolysates provide highly digestible low-molecular-weight peptides and free amino acids, enhancing palatability, digestive efficiency, and immune competence in line with shrimp feeding ecology [7,16,17,18,33,34]. Accordingly, the present study demonstrates that 1% tuna hydrolysate is an effective partial substitute for squid liver powder, supporting growth, digestion, and health while complementing plant-based formulations.

## 5. Conclusions

Based on the present findings, an inclusion level of 1% tuna hydrolysate liquid (TH, dry matter basis) was identified as optimal for Pacific white shrimp when formulated with 10% fish meal. Replacing 1% of the diet with TH resulted in significantly improved growth performance, feed utilization, and survival compared with the other hydrolysate-based diets. TH replacement also supported digestive function through increased trypsin activity and higher protein digestibility and promoted favorable histological characteristics in hepatopancreatic and intestinal tissues. From an immunological perspective, shrimp fed the TH diet exhibited elevated lysozyme and phenol oxidase activities and greater hemolymph inhibitory activity against *Vibrio parahaemolyticus*. Overall, these findings support the use of tuna hydrolysate liquid as a functional and sustainable alternative to squid-liver powder in shrimp diets.

## Figures and Tables

**Table 1:** Chemical composition, molecular-weight, and amino acid profile of squid-liver powder (SLP), tuna hydrolysate liquid (TH), shrimp hydrolysate powder (SH), fish hydrolysates powder (FH) and salmon silage liquid (SS), based on technical data sheets.

	Ingredients				
	SLP	TH	SH	FH	SS
**Chemical composition (% wet basis)**					
Moisture	7.8	50.4	4.4	5.8	54.9
Crude protein	44.7	32.5	68.1	82.4	31.0
Crud lipid	17.1	2.1	8.9	7.75	8.6
Ash	7.2	11.4	11.8	3.9	3.0
Energy (MJ kg^−1^)	22.42	14.10	20.88	22.81	24.22
Soluble protein	n.d. *	>90	>90	>90	n.d. *
**Molecular weight (Dalton, % total protein)**					
>10,000	n.d. *	2	0	1	0
5000–10,000	n.d. *	7	1	1	1
1000–5000	n.d. *	26	10	11	9
500–1000	n.d. *	9	10	14	7
<500	n.d. *	54	79	73	82
**Essential amino acids (% wet basis)**					
Arginine	2.05	1.66	4.19	4.04	1.97
Histidine	2.43	1.59	1.30	1.73	0.46
Isoleucine	2.43	0.54	2.70	3.05	1.20
Leucine	3.16	1.11	4.40	5.27	2.31
Lysine	2.04	1.36	4.30	5.07	2.40
Methionine	0.80	0.44	1.30	1.92	0.95
Phenylalanine	2.06	0.60	2.90	2.84	1.17
Threonine	1.34	0.86	2.50	3.54	1.17
Tryptophan	0.65	0.01	0.80	0.65	0.37
Valine	2.19	0.75	3.20	3.47	1.51
Total essential amino acids	19.14	8.92	27.59	31.58	13.52
**Non-essential amino acids (% wet basis)**					
Alanine	1.35	2.04	4.30	5.40	1.85
Aspartic acid	1.94	1.60	5.70	6.48	2.86
Glutamic acid	2.69	2.88	9.00	10.35	4.31
Glycine	1.37	3.62	4.90	6.78	1.85
Tyrosine	1.53	0.37	2.30	2.05	1.05
Serine	0.83	0.91	2.40	3.08	1.85
Total non-essential amino acids	15.29	13.56	32.21	39.11	13.98
Total amino acids	34.43	22.48	59.80	70.69	27.50

Abbreviations: SLP, squid-liver powder; TH, tuna hydrolysate liquid; SH, shrimp hydrolysate powder; FH, fish hydrolysate powder; and SS, salmon silage liquid. * n.d. = no data.

**Table 2:** Formulation and chemical composition of the experimental diets for Pacific white shrimp (% fed basis).

Ingredients	Dietary Treatment				
	SLP	TH	SH	FH	SS
Fish meal, 65% CP	10.00	10.00	10.00	10.00	10.00
Poultry by-product meal	8.00	8.00	8.00	8.00	8.00
Soybean meal, 48%CP	27.52	30.05	30.75	30.11	30.00
Soy protein concentrate	10.00	10.00	10.00	10.00	10.00
Wheat gluten meal, 78% CP	5.00	5.00	5.00	5.00	5.00
Wheat flour	24.60	24.24	24.34	25.16	24.11
Tuna crude oil	1.81	1.80	1.81	1.81	1.79
Soybean oil	1.20	1.76	1.76	1.76	1.61
Lecithin	0.82	1.00	1.00	1.00	1.00
Choline chloride, 60% choline	0.50	0.50	0.50	0.50	0.50
Cholesterol	0.12	0.12	0.12	0.12	0.12
MCP ^1^	1.63	1.52	1.70	1.52	1.70
Stay C-35 ^2^	0.50	0.50	0.50	0.50	0.50
DL-Methionine	0.00	0.06	0.06	0.04	0.05
L-Lysine	0.00	0.13	0.11	0.12	0.10
Salt, NaCl	0.60	0.60	0.60	0.60	0.60
Vitamin premix ^3^	0.50	0.50	0.50	0.50	0.50
Mineral premix ^4^	0.50	0.50	0.50	0.50	0.50
Guar gum	1.50	1.50	1.50	1.50	1.50
Antioxidant agents ^5^	0.10	0.10	0.10	0.10	0.10
Antimicrobial agents ^6^	0.10	0.10	0.10	0.10	0.10
Squid-liver Powder	5.00				
Tuna hydrolysate liquid		2.02			
Shrimp hydrolysate powder			1.05		
Fish hydrolysate powder				1.06	
Salmon silage liquid					2.22
**Analyzed chemical composition (% dry matter) ^7^**					
Dry matter	92.29	91.67	92.04	93.27	93.17
Crude protein	44.31	44.10	44.31	44.18	44.06
Crude lipid	9.40	9.71	9.78	9.43	9.55
Crude fiber	2.75	2.36	2.54	2.49	2.21
Ash	11.56	11.61	11.47	11.35	11.45
NFE	23.27	23.89	23.94	25.82	25.90
Gross energy (MJ kg^−1^)	19.75	19.70	19.85	19.82	19.66

Abbreviations: CP, crude protein; SLP, squid-liver powder; TH, tuna hydrolysate liquid; SH, shrimp hydrolysate powder; FH, fish hydrolysate powder; and SS, salmon silage liquid. ^1^ Mono Calcium Phosphate (Ca(H_2_PO_4_)2H_2_O. ^2^ Stay C-35 ascorbyl mono phosphate (1 kg), 350,000 mg kg^−1^, DMS. ^3^ Vitamin premix (1 kg) contains 0.9 g vitamin A, 1.5 g vitamin B1-Thiamin, 12.5 g vitamin B2-Riboflavin, 50 g vitamin B3-Niacin, 40 g vitamin B5-Pantothenic acid, 20 g vitamin B6-Pyridoxine, 0.75 g vitamin B7-Biotin, 3 g vitamin B9-Folic acid, 0.1 g vitamin B12, 0.025 g vitamin D, 90 g vitamin E, and 20 g vitamin K. ^4^ Mineral premix (1 kg) contains 0.15 g Ca, 0.15 g P, 20 g Cu, 40 g Fe, 15 g Mn, 280 mg Se, 40 g Zn, and 2.2 g I. ^5^ Antioxidant agents, Butylated Hydroxytoluene (C_15_H_24_O). ^6^ Antimicrobial agents, Calcium propanoate (Ca(C_2_H_5_COO)_2_). ^7^ Chemical analyses were performed at the Agricultural Development Research Center, Faculty of Agriculture, Khon Kaen University, using standard AOAC methods (dry matter 934.01; crude protein Kjeldahl; crude lipid 2003.05; fiber Fibertherm method ash 942.05). Nitrogen-free extract (NFE) was calculated as the percentage of dry matter minus the sum of crude protein, crude lipid, crude ash, and crude fiber [26]. Gross energy was measured using an automatic adiabatic bomb calorimeter (IKA^®^ Werke, C5000, Staufen, Germany).

**Table 3:** Molecular-weight and amino acids profiles of the experimental diet for Pacific white shrimp.

Parameters	Dietary Treatments				
	SLP	TH	SH	FH	SS
**Molecular** **-** **weight (Dalton, area % of detected peptides) ^1^**					
>20,000	21.1	20.1	18.3	17.7	18.6
10,000–20,000	7.6	8.2	6.7	7.2	6.8
5000–10,000	3.2	3.8	2.8	3.1	2.7
1000–5000	4.9	5.9	4.8	5.9	4.5
500–1000	2.5	2.6	2.9	3.3	2.7
<500	60.7	59.4	64.5	62.8	64.7
**Essential amino acid (% dry matter) ^2^**					
Arginine	2.61	2.70	2.61	2.48	2.47
Histidine	1.08	1.19	1.14	1.07	1.09
Isoleucine	1.81	1.89	1.85	1.77	1.77
Leucine	2.87	2.97	2.89	2.80	2.80
Lysine	2.42	2.52	2.44	2.39	2.41
Methionine	0.68	0.73	0.71	0.64	0.65
Phenylalanine	1.87	1.97	1.94	1.90	1.90
Threonine	1.50	1.54	1.53	1.45	1.44
Tryptophan	0.36	0.45	0.38	0.35	0.34
Valine	2.40	2.47	2.40	2.41	2.42
**Non-essential amino acid (%, dry matter) ^2^**					
Alanine	1.82	1.90	1.91	1.79	1.79
Aspartic acid	4.07	3.78	3.80	3.73	3.68
Cystine + Cysteine	0.56	0.57	0.68	0.61	0.61
Glutamic acid	8.84	7.73	8.09	8.00	8.08
Glycine	2.15	2.28	2.16	1.99	2.10
Proline	2.60	2.73	2.59	2.38	2.59
Taurine	0.05	0.07	0.06	0.04	0.04
Tyrosine	1.35	1.45	1.44	1.40	1.38
Serine	1.83	1.84	1.84	1.71	1.71
Total essential amino acids	17.74	18.54	18.00	17.43	17.45
Total non-essential amino acids	23.29	22.35	22.56	21.65	21.98
Total amino acids	41.02	40.89	40.55	39.08	39.42

Abbreviations: SLP, squid-liver powder; TH, tuna hydrolysate liquid; SH, shrimp hydrolysate powder; FH, fish hydrolysate powder; and SS, salmon silage liquid. ^1^ Molecular-weights were analyzed following water-soluble extraction; therefore, insoluble intact proteins were excluded from the analysis. The molecular weight distribution of peptides was determined using size-exclusion chromatography coupled with high-performance liquid chromatography (SEC-HPLC). Values are expressed as the area percentage of detected peptide fractions. ^2^ Amino acid composition was determined by SGS Thailand following ISO 13903 using HPLC [36].

**Table 4:** Growth performance and feed utilization of Pacific white shrimp fed the experimental diets over an 8-week feeding period.

Parameters	Dietary Treatments					*p*-Value
	SLP	TH	SH	FH	SS	
IBW (g shrimp^−1^) ^1^	2.66 ± 0.01	2.66 ± 0.01	2.66 ± 0.01	2.66 ± 0.01	2.66 ± 0.01	0.921
FBW (g shrimp^−1^) ^2^	14.69 ± 0.46 ^b^	15.76 ± 0.24 ^a^	15.05 ± 0.40 ^b^	14.79 ± 0.56 ^b^	14.57 ± 0.37 ^b^	0.002
SGR (% day^−1^) ^3^	3.05 ± 0.06 ^b^	3.18 ± 0.02 ^a^	3.10 ± 0.05 ^b^	3.06 ± 0.07 ^b^	3.04 ± 0.05 ^b^	0.003
FI (g shrimp^−1^) ^4^	19.49 ± 1.45 ^b^	20.96 ± 0.49 ^a^	19.93 ± 0.71 ^ab^	19.11 ± 0.69 ^b^	20.07 ± 0.43 ^ab^	0.026
FCR ^5^	1.58 ± 0.15	1.51 ± 0.05	1.53 ± 0.06	1.53 ± 0.03	1.61 ± 0.05	0.311
HSI (%) ^6^	4.35 ± 0.32	4.84 ± 0.84	4.69 ± 0.82	4.47 ± 0.78	4.79 ± 0.90	0.819
Survival rate (%)	86.8 ± 3.6 ^a^	90.0 ± 2.8 ^a^	87.2 ± 1.1 ^a^	81.6 ± 3.0 ^b^	83.2 ± 1.1 ^b^	<0.001

Note: Values are the means of five replicate groups and are presented as mean ± standard deviation. Values with different superscripts in the same row are significantly different (*p* < 0.05). Dietary treatments are abbreviated: SLP, squid-liver powder; TH, tuna hydrolysate liquid; SH, shrimp hydrolysate powder; FH, fish hydrolysate powder; and SS, salmon silage liquid. ^1^ IBW, Initial body weight; ^2^ FBW, Final body weight; ^3^ SGR, Specific growth rate; ^4^ FI, Feed intake; ^5^ FCR, Feed conversion ratio; and ^6^ HSI, Hepatosomatic index.

**Table 5:** Metabolic markers of Pacific white shrimp hemolymph fed the experimental diets for 8 weeks.

Parameters	Dietary Treatment					
	SLP	TH	SH	FH	SS	*p*-Value
Cholesterol (mg dL^−1^)	1.1 ± 0.2	1.1 ± 0.2	1.8 ± 1.5	1.2 ± 0.4	1.2 ± 0.3	0.536
Triglyceride (mg dL^−1^)	56.6 ± 13.8	65.5 ± 15.7	60.4 ± 13.8	57.5 ± 11.5	66.0 ± 11.2	0.705
Albumin (g dL^−1^)	1.2 ± 0.2	1.3 ± 0.1	1.4 ± 0.2	1.3 ± 0.2	1.3 ± 0.1	0.579
Total protein (g dL^−1^)	5.5 ± 1.3	6.2 ± 0.8	6.7 ± 0.6	6.0 ± 1.1	6.0 ± 0.7	0.393
Glucose (mg dL^−1^)	8.3 ± 1.4 ^b^	10.2 ± 1.8 ^b^	8.5 ± 4.2 ^b^	14.9 ± 3.9 ^a^	10.5 ± 0.9 ^b^	0.009
Creatinine (mg dL^−1^)	0.5 ± 0.2	0.7 ± 0.1	0.6 ± 0.2	0.5 ± 0.3	0.8 ± 0.2	0.101
Aspartate transaminase (U L^−1^)	28.1 ± 12.2	31.1 ± 5.6	30.8 ± 4.6	53.0 ± 23.5	34.9 ± 11.7	0.052
Alkaline phosphatase (U L^−1^)	107.9 ± 142.3	69.3 ± 31.3	55.4 ± 26.7	131.9 ± 29.0	85.6 ± 31.0	0.441

Note: Values are the means of five replicate groups and are presented as mean ± standard deviation. Values with different superscripts in the same row are significantly different (*p* < 0.05). Dietary treatments are abbreviated: SLP, squid-liver powder; TH, tuna hydrolysate liquid; SH, shrimp hydrolysate powder; FH, fish hydrolysate powder; and SS, salmon silage liquid.

**Table 6:** Digestive enzyme activity in hepatopancreas of Pacific white shrimp fed the experimental diets for 8 weeks.

Digestive Enzyme Activity (unit h^−1^ mg protein^−1^)	Dietary Treatment					*p*-Value
	SLP	TH	SH	FH	SS	
Trypsin activity	2343 ± 525 ^b^	3236 ± 226 ^a^	2702 ± 332 ^ab^	2503 ± 605 ^b^	2494 ± 387 ^b^	0.035
Chymotrypsin activity	1090 ± 257	1308 ± 209	1060 ± 151	1018 ± 113	998 ± 127	0.085
Protease activity	468 ± 54	461 ± 138	439 ± 63	484 ± 99	461 ± 35	0.947
Amylase activity	1440 ± 306	1395 ± 274	1394 ± 238	1330 ± 258	1350 ± 104	0.960
Lipase activity	477 ± 152	469 ± 136	475 ± 135	395 ± 127	352 ± 86	0.451

Note: Values are the means of five replicate groups and are presented as mean ± standard deviation. Values with different superscripts in the same row are significantly different (*p* < 0.05). Dietary treatments are abbreviated: SLP, squid-liver powder; TH, tuna hydrolysate liquid; SH, shrimp hydrolysate powder; FH, fish hydrolysate powder; and SS, salmon silage liquid.

**Table 7:** Apparent digestibility coefficients (%, ADC) of dry matter, protein, and lipid of the experimental diets for Pacific white shrimp.

Parameters	Dietary Treatments					
	SLP	TH	SH	FH	SS	*p*-Value
ADC_d_ ^1^	82.3 ± 2.1	83.7 ± 0.5	83.3 ± 1.6	82.7 ± 2.7	80.4 ± 1.6	0.261
ADC_p_ ^2^	87.4 ± 0.4 ^d^	91.4 ± 0.4 ^a^	90.5 ± 0.5 ^b^	88.5 ± 0.1 ^c^	87.6 ± 0.2 ^d^	<0.001
ADC_l_^3^	94.6 ± 0.6	94.4 ± 0.2	93.6 ± 0.7	94.3 ± 0.9	94.0 ± 0.2	0.350

Note: Values are the means of five replicate groups and are presented as mean ± standard deviation. Values with different superscripts in the same row are significantly different (*p* < 0.05). Dietary treatments are abbreviated: SLP, squid-liver powder; TH, tuna hydrolysate liquid; SH, shrimp hydrolysate powder; FH, fish hydrolysate powder; and SS, salmon silage liquid. ^1^ ADC_d_: Apparent digestibility coefficient of dry matter, ^2^ ADC_p_: Apparent digestibility coefficient of protein, ^3^ ADC_l_: Apparent digestibility coefficient of lipid.

## Data Availability

Additional data supporting the findings of this study are available from the corresponding authors upon reasonable request. Raw data and histological images used in this study can be accessed upon request.
